# Overexpression of TRIM14 promotes tongue squamous cell carcinoma aggressiveness by activating the NF-κB signaling pathway

**DOI:** 10.18632/oncotarget.6941

**Published:** 2016-01-18

**Authors:** Xuan Su, Jianning Wang, Weichao Chen, Zhaoqu Li, Xiaoyan Fu, Ankui Yang

**Affiliations:** ^1^ Department of Head and Neck Surgery, Sun Yat-sen University Cancer Center, State Key Laboratory of Oncology in South China, Collaborative Innovation Center for Cancer Medicine, Guangzhou, Guangdong, 510060, P. R. China; ^2^ Department of Oral and Maxillofacial Surgery, Guanghua School of Stomatology, Institute of Stomatological Research, Sun Yat-sen University, Guangdong Provincial Key Laboratory of Stomatology, Guangzhou, Guangdong, 510055, China

**Keywords:** TRIM14, TSCC, aggressiveness, NF-κB pathway

## Abstract

Tongue squamous cells carcinoma (TSCC) is one of the most lethal malignancies of oral cancers and its prognosis remains dismal due to the paucity of effective therapeutic targets. Herein, we showed that Tripartite motif containing 14(TRIM14) is markedly up-regulated in TSCC cell lines and clinical tissues. Immunohistochemical (IHC) analysis of 116 clinical TSCC specimens revealed that TRIM14 expression was significantly correlated with the TNM classification (T: P = 0.01; N: P < 0.001; M: P < 0.001) in patients with TSCC. Multivariate analysis indicated that TRIM14 expression might be an independent prognostic indicator for the survival of patients with TSCC. Ectopic expression of TRIM14 in TSCC cells promoted proliferation, angiogenesis, and increased resistance to cisplatin-induced apoptosis of TSCC cells *in vitro*. Furthermore, TRIM14 overexpressing significantly promoted the tumorigenicity of TSCC cells *in vivo* whereas silencing endogenous TRIM14 caused an opposite outcome. Moreover, we demonstrated that TRIM14 enhanced TSCC aggressiveness by activating NF-κB signaling. Together, our results provide new evidence that TRIM14 overexpression promotes the progression of TSCC and might represent a novel therapeutic target for its treatment.

## INTRODUCTION

Oral squamous cell carcinoma (OSCC) represents the sixth most common solid cancer worldwild and tongue squamous carcinoma (TSCC) is one of the major oral malignant tumor subtypes [1-3]. The main curative treatments for TSCC are the surgical removal of tongue lesions, radiotherapy and chemotherapy [4]. Although the clinical treatment outcomes for tongue cancer have improved in recent decades, the overall 5-year survival rate for TSCC patients is only 50% due to lacking of new diagnostic and treatment methods [5, 6]. Therefore, it is of great clinical value to explore the molecular mechanisms in development of TSCC and identify effective treatment strategies to improve the survival rate for TSCC patients.

Accumulating evidences suggest that poor therapeutic effect and the dismal survival rate of TSCC are associated with aberrantly activated signaling pathways, including nuclear factor-κB (NF-κB) signaling [7]. It has been reported that the constitutive activation of NF-κB signaling plays key roles in the development and progression of TSCC and contributes to characteristics of the malignant phenotype in TSCC [8-11]. For instance, Peng *et al.* found that activated NF-κB signaling results in TSCC cell resistance to chemotherapy and promotes cell survival, while inhibition of NF-κB signaling dramatically reduces the proliferation of oral squamous cell carcinoma [11]. Wang and colleague showed that NF-κB signaling is involved in the EGF-induced EMT and is positively associated with lymph node metastasis of TSCC [8]. Conversely, blockade of NF-κB signaling contributed the antitumor activity of Trichostatin A in human tongue carcinoma cells [9] and the down-regulation of NF-κB p65 can inhibit invasion and migration of human tongue cancer SCC4 cells induced by gypenosides [10]. Therefore, discovering novel molecular(s) that can regulate aberrant activation of the NF-κB pathway could be important for clinical TSCC therapy.

Tripartite motif containing 14 (TRIM14), a newly identified gene located on chromosome 9q22, contains a B-box, a coiled-coil domain, and a C-terminal PRYSPRY domain but lacks the N-terminal RING domain found in most TRIM family proteins [12]. Previously, Valentina *et al.* found that TRIM14 overexpression in human HEK293 cell resulted in the up-regulation of many genes, including *Tnfrsf13c*, *Hbp1* and *Pdgfrb*, which have been reported to be involved in the activation of NF-κB and Wnt/β-catenin signaling pathways [13]. High expression levels of TRIM14 have also been reported in human immunodeficiency virus (HIV)-associated human and simian immunodeficiency virus (SIV)-associated monkey lymphomas [14]. Furthermore, enhanced expression of TRIM14 gene can suppress Sindbis virus reproduction by increasing the transcription of many genes involved in innate immunity [15]. Importantly, Zhou and colleague demonstrated that TRIM14 acts as a mediator in immune responses against viral infection by activating NF-κB pathways by recruiting NF-κB essential modulator (NEMO) to the MAVS signalosome [16]. Therefore, these results suggest that upreguation of TRIM14 might play a role in activation of the NF-κB pathway and in tumorigenesis.

In this current study, we reported that TRIM14 expression was significantly upregulated in tongue cancer cells and clinical tissues, and was associated with the clinical features of tongue cancer. Overexpression of TRIM14 could promote the proliferation, metastasis, angiogenesis and resistance to apoptosis of TSCC cells. Our findings suggest that TRIM14 plays critical oncogenic role in TSCC progression and highlights its potential as a target for TSCC therapy.

## RESULTS

### Overexpression of TRIM14 correlates with TSCC progression and poor prognosis

**Figure 1:**
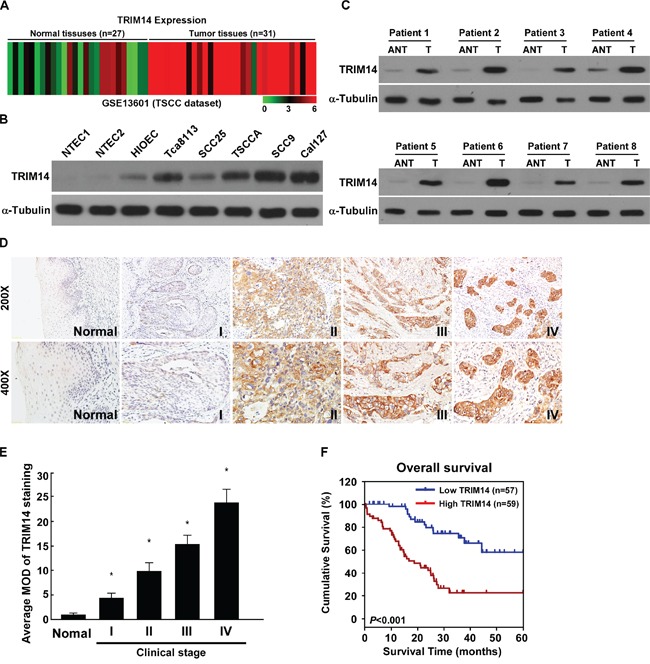
Overexpression of TRIM14 correlates with TSCC progression and poor prognosis **A.** Expression profiling of mRNAs showing that TRIM14 is upregulated in TSCC tissues (T) compared to normal tissues (n =58; NCBI/GEO/GSE13601). **B-C.** Western blotting analysis of TRIM14 expression in one immortalized oral epithelial cell line (HIOEC) and five cultured TSCC cell lines (B) as well as in eight paired human TSCC tissues (T) and the matched adjacent non-tumor tissues (ANT) from the same patient (C) α-Tubulin was used as a loading control. **D.** IHC staining indicating the TRIM14 protein expression in human TSCC (clinical stage I-IV) compared with adjacent tongue tissues. **E.** The average MOD of TRIM14 staining between adjacent tongue tissues and different clinical stage TSCC were statistically quantified. **F.** The Kaplan-Meier survival curves compare TSCC patients with low and high TRIM14 expression levels (n = 116; *P* < 0.001).

By analyzing the published mRNA expression profiles obtained from 31 TSCC tissues and 27 normal tongue tissues (NCBI/GEO/GSE13601), we found that *TRIM14* was significantly upregulated in TSCC tissues compared with normal tissues (Figure 1A). Consistently, real-time PCR and western blotting analyses revealed that TRIM14 was markedly overexpressed in all five TSCC cell lines and HIOEC immortalized oral epithelial cells, at both the protein and mRNA levels, compared with two normal tongue epithelial cells NTECs (Figure 1B and Supplementary Figure 1A). Furthermore, comparative analyses showed that TRIM14 expression were elevated in the ten TSCC samples compared with matched adjacent non-tumor tissues (Figure 1C and Supplementary Figure 1B), suggesting that TRIM14 is upregulated in human TSCC.

To determine the clinical relevance of TRIM14 in TSCC, TRIM14 expression was examined in 116 paraffin-embedded, archived TSCC tissues by IHC assay. As showed in Figure 1D and Supplementary Table 2, TRIM14 levels were correlated with the clinical stage (*P* = 0.009), and TNM classification (T: *P* = 0.01; N: *P* < 0.001; M: *P* < 0.001) in patients with TSCC. Quantitative analysis indicated that the average MODs of TRIM14 staining in clinical stage I–IV primary tumors were significantly higher than those in adjacent non-cancerous tissues (*P* <0.001, Figure 1E). Importantly, patients with higher TRIM14 expression exhibited shorter survival and patients with lower TRIM14 expression had longer survival (*P* < 0.001 Figure 1F). Moreover, univariate and multivariate analyses indicated that TRIM14 expression was an independent prognostic factor in TSCC (Supplementary Table 3). Collectively, our findings suggest a potential association between TRIM14 upregulation and TSCC progression.

### Up-regulation of TRIM14 promotes the aggressiveness of TSCC cells *in vitro*

**Figure 2:**
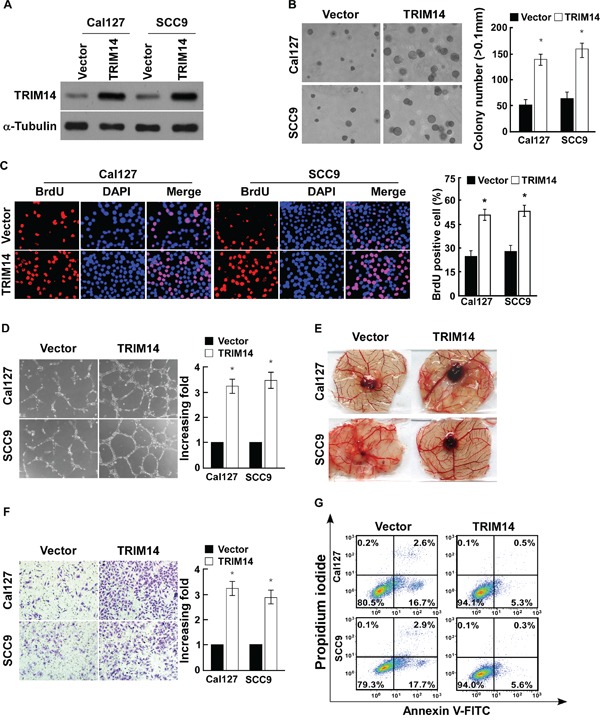
Up-regulation of TRIM14 expression promotes TSCC cell aggressiveness *in vitro* **A.** Western blot analysis of TRIM14 expression in the indicated cells. α-Tubulin was used as a loading control. **B.** Representative pictures (left panel) and quantification (right panel) of the colony numbers of indicated cells as determined using an anchorage-independent growth assay. Colonies larger than 0.1 mm in diameter were scored. **C.** Representative micrographs (left) and quantification of BrdU positive signaling in the cells transfected with TRIM14 or vector. **D.** Representative images (left panel) and quantification (right panel) of HUVECs cultured on matrigel-coated plates with conditioned medium from vector control and TRIM14-transduced TSCC cells. **E.** Representative images of CAM blood vessels stimulated with conditioned medium from the indicated cells. **F.** Representative pictures (left panel) and quantification (right panel) of invaded cells were analyzed using a transwell matrix penetration assay. **G.** Annexin V-FITC and PI staining of the indicated cells treated with cisplatin (20 μM) for 24 h. Each bar represents the mean ± SD of three independent experiments. * *P* <0.05.

To investigate the biological role of TRIM14 up-regulation in TSCC progression, Cal127 and SCC9 cell lines that stably expressed TRIM14 were established (Figure 2A). We found that ectopic expression of TRIM14 in TSCC cells markedly increased the growth rate and augmented the anchorage-independent growth ability in these cells (Figure 2B-2C). Meanwhile, the ability of TSCC cells to induce HUVEC tube formation and CAM neovascularization, and invasive ability of TSCC cells was significantly increased in the TSCC cells that overexpressed TRIM14 (Figure 2D-2F). Furthermore, overexpression of TRIM14 could also enhance the resistance of TSCC cells to apoptosis induced by treatment with the chemotherapeutic agent cisplatin (Figure 2G). The oncogenic role of TRIM14 was also observed in HIOEC cells (Supplementary Figure 2). Collectively, these results suggest that TRIM14 upregulation promotes the aggressiveness of TSCC cells *in vitro*.

### Down-regulation of TRIM14 suppresses the aggressiveness of TSCC cells *in vitro*

**Figure 3:**
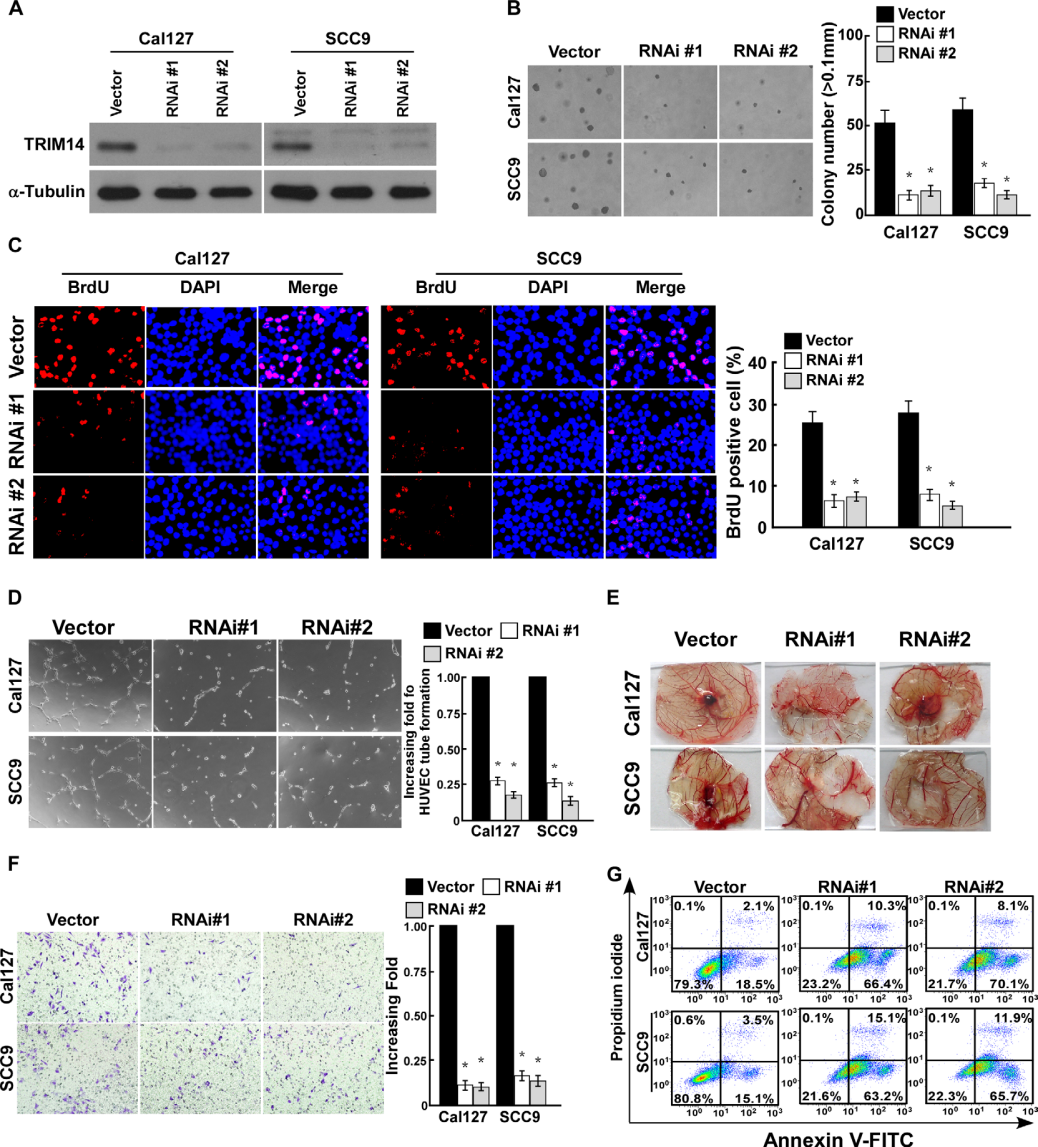
Downregulation of TRIM14 suppresses the aggressiveness of TSCC cells **A.** Western blot analysis of TRIM14 expression in the indicated cells. α-Tubulin was used as a loading control. **B.** Representative pictures of (left panel) and quantification (right panel) of colony numbers of indicated cells as determined by an anchorage-independent growth assay. Colonies larger than 0.1 mm in diameter were scored. **C.** Representative micrographs (left) and quantification of BrdU positive signaling in the cells transfected with TRIM14-RNAi or RNAi-vector. **D.** Representative images (left panel) and quantification (right panel) of HUVECs cultured on matrigel-coated plates with conditioned medium from control and TRIM14-RNAi TSCC cells. **E.** Representative images of the CAM blood vessels stimulated with conditioned medium from indicated cells. **F.** Representative pictures of (left panel) and quantification (right panel) of invaded cells were analyzed using a transwell matrix penetration assay. **G.** Annexin V-FITC and PI staining of indicated cells treated with cisplatin (20 μM) for 24 h. Each bar represents the mean ± SD of three independent experiments. * *P* <0.05.

The downregulation of TRIM14 in Cal127 and SCC9 cell lines dramatically inhibited the proliferative capacity of TSCC cells (Figure 3A-3C). Additionally, downregulation of TRIM14 decreased TSCC cells invasive ability and repressed the ability of TSCC cells to induce HUVEC tube formation and CAM neovascularization, and increased the sensitivity of TSCC cells to cisplatin (Figure 3D-3G). Together, these data suggest that downregulation of TRIM14 inhibits TSCC aggressiveness *in vitro.*

### Over-expression of TRIM14 contributes to TSCC progression *in vivo*

**Figure 4:**
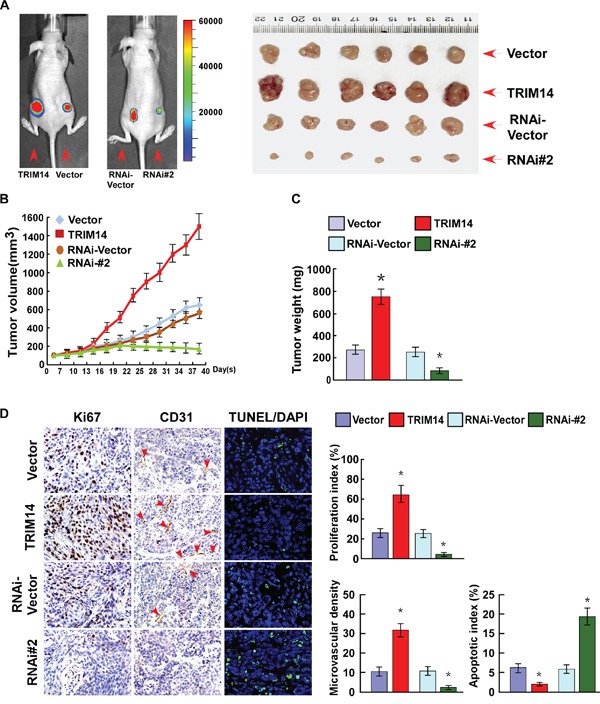
Overexpression of TRIM14 contributes to TSCC progression *in vivo* **A.** Representative images of tumor-bearing mice (left) and tumors from all of the mice in each group (right). **B.** Tumor volumes were measured on the indicated days. **C.** Mean tumor weights. **D.** IHC staining demonstrated the expression of Ki67 and CD31 as well as TUNEL-positive cells in the indicated tissues.

The biological effects of TRIM14 overexpression on TSCC progression were further examined using a xenograft tumor model. As showed in Supplementary Figure 3 and Figure 4A-4C, tumors formed by TRIM14-overexpressing cells exhibited a greater size and mass than tumors formed by the control cells. Conversely, tumors formed by TRIM14-silenced TSCC cells were smaller and had lower tumor weights than control tumors. IHC analysis revealed that TRIM14-overexpressing tumors showed increased percentages of Ki67-positive cells, greater microvascular density (MVD) and fewer TUNEL-positive cells, whereas TRIM14-silenced tumors displayed lower Ki67 proliferation index and MVD and a higher percentage of TUNEL-positive apoptotic cells (Figure 4D). Taken together, our findings indicate that TRIM14-overexpression contributes to TSCC progression *in vivo*.

### TRIM14 upregulation activates the NF-κB signaling pathway in TSCC

**Figure 5:**
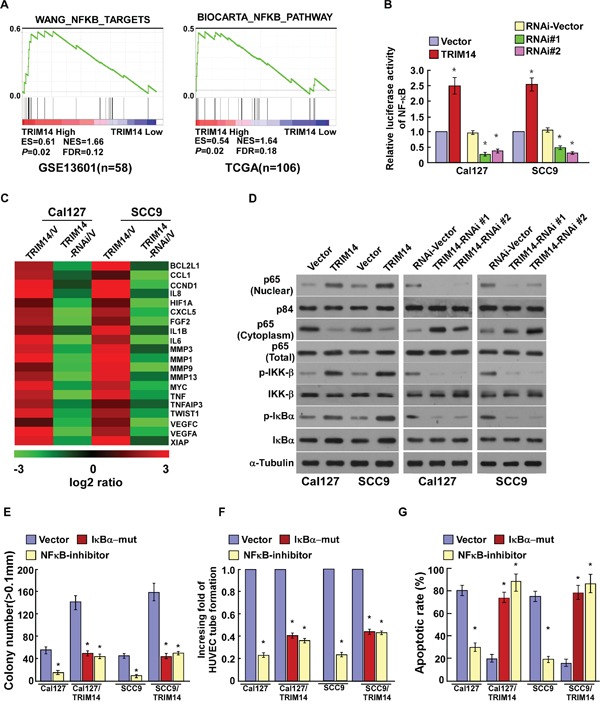
TRIM14 up-regulation activates the NF-κB signaling pathway in TSCC **A.** GSEA plot, indicating a significant correlation between the mRNA levels of TRIM14 expression in TSCC and the NF-κB-activated gene signatures in published datasets. **B.** Analysis of luciferase reporter activity in the indicated cells after transfection with 100 ng pNF-κB–luc plasmids or control-luciferase plasmid. **C.** Real-time PCR analysis demonstrating an apparent overlap between NF-κB–dependent gene expression and TRIM14–regulated gene expression. The pseudo color represents an intensity scale for TRIM14 versus vector or TRIM14 siRNA versus control siRNA, calculated by log2 transformation. **D.** Western blotting analysis of the expression levels of the indicated proteins in the indicated cells.α-tubulin was used as a loading control. **E.** Quantification of colony numbers as determined by anchorage-independent growth assay. Colonies larger than 0.1 mm in diameter were scored. **F.** Quantification of tubule formation by HUVECs cultured in Matrigel-coated plates with conditioned media from TSCC cells transfected with the vector, IκBα-mut or treated with the NF-κB inhibitor (JSH-23). **G.** Quantification of cisplatin-induced (20μM) TUNEL-positive cells in TSCC cells transfected with vector, IκBα-mut or treated with the NF-κB inhibitor. Each bar represents the mean ± SD of three independent experiments; *P < 0.05.

By analyzingTRIM14 mRNA expression levels and NF-κB-regulated gene signatures from published TSCC patient profiles (NCBI/GEO/GSE13601 and TCGA/TSCC datasets), we found that TRIM14 expression was positively correlated with NF-κB signaling gene signatures (Figure 5A), suggesting that TRIM14 might be involved in regulation of the NF-κB signaling pathway in TSCC. As expected, overexpression of TRIM14 significantly enhanced, whereas silencing of TRIM14 reduced, the activity of NF-κB luciferase reporter activity (Figure 5B). Furthermore, the expression levels of numerous well-characterized NF-κB downstream genes were showed to be increased in TRIM14 overexpressing cells, but were lower in TRIM14-silenced cells (Figure 5C). Moreover, western blotting revealed that the levels of nuclear p65 and phosphorylated-IKK-β and -IκBα were dramatically upregulated in TRIM14-overexpressing cells but were downregulated in TRIM14-silenced cells (Figure 5D and Supplementary Figure 4), suggesting that TRIM14 plays an important role in activating the NF-κB signaling pathway.

Next, we investigated whether TRIM14-mediated TSCC progression occurs through NF-κB activation. As shown in Supplementary Figure 5, the stimulatory effect of TRIM14 on NF-κB activation was significantly inhibited by transfection of an IκBα dominant-negative mutant (IκBα-mut) or treatment with a NF-κB inhibitor. Meanwhile, we found that blockade of the NF-κB pathway significantly abrogates the effect of TRIM14 on TSCC aggressiveness in both *in vitro* and *in vivo* (Figure 5E-5G and Supplementary Figure 6). Taken together, these results indicate that activation of the NF-κB signaling pathway exerted functional effects of TRIM14 on TSCC progression.

### Clinical relevance of TRIM14-induced NF-κB activation in human TSCC

**Figure 6:**
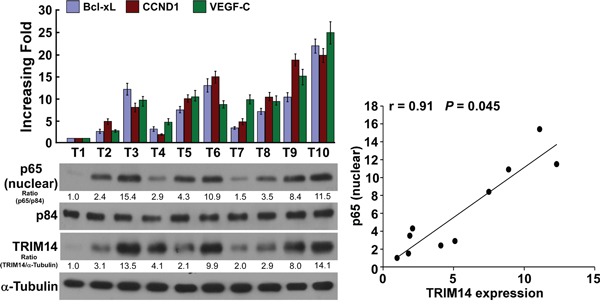
Clinical relevance of TRIM14-induced NF-κB activation in human TSCC Expression analysis (left) and correlation (right) of TRIM14 expression and BCL-xL, CCND1, VEGF-C and nuclear p65 expression in 10 freshly collected human TSCC tissue samples (T); α-Tubulin and nuclear protein p84 were used as loading controls. Each bar represents the mean ± SD of three independent experiments.

The clinical relevance of TRIM14 expression and NF-κB activation was further characterized in human TSCC. As showed in Figure 6 and Supplementary Table 4, TRIM14 levels in ten freshly collected clinical TSCC samples were positively correlated with nuclear p65 signals (r = 0.91, *P* = 0.045) and the mRNA expression of NF-κB downstream genes: Bcl-xL (r = 0.90, *P* = 0.045), CCND1 (r = 0.76, *P* = 0.036) and VEGF-C (r = 0.71, *P* = 0.045). These data further support the notion that TRIM14 up-regulation promotes TSCC aggressiveness and activation of the NF-κB signaling pathway, which may lead to a poor clinical outcome for patients with TSCC.

## DISCUSSION

In the current study, we found that TRIM14 might play an important role in the malignant progression of TSCC and in regulation of the NF-κB signaling pathway. IHC analysis revealed that TRIM14 was significantly upregulated in TSCC and was associated with the clinical features and poor overall survival of TSCC patients. Overexpression of TRIM14 augmented the anchorage-independent growth and the invasive abilities of TSCC cells, provoked their ability to induce HUVEC tube formation and CAM neovascularization and enhanced their resistance to apoptosis. These findings provide novel insights into the potential roles of TRIM14 deregulation in promoting carcinogenesis and progression of TSCC.

It has been well established that NF-κB transcription factors not only orchestrate immune and inflammatory responses, but also plays a crucial role in oncogenesis [17, 18]. Therapeutic targeting of the NF-κB pathway has been aggressively pursued for the treatment of a wide range of inflammatory and malignant pathologies, including TSCC [8-10, 19]. Wang *et al.* demonstrated that NF-κB can regulate EGF-induced EMT and cancer metastasis, but NF-κB inhibitors could block EGF-induced EMT, and suppress the invasion and migration of TSCC [8]. Additionally, Trichostatin A treatment could significantly suppress NF-κB activity and induce Tca8113 cell apoptosis by promoting the up-regulation of the pro-apoptotic protein Bax and downregulation of the anti-apoptotic proteins Bcl-2 and Bcl-xL [9]. Moreover, Kung and colleagues reported that down-regulation of NF-κB by gypenosides could inhibits invasion and migration of human tongue SCC4 cell [10]. Collectively, these findings establish a strong rationale for therapeutic targeting of the NF-κB pathway in TSCC. Nevertheless, achieving improved treatment outcomes has been an insurmountable challenge to date. Although current therapeutic approaches, such as the use of NF-κB or IKK-β inhibitors, could potentially abrogate the cancer-promoting activities of NF-κB, they fail to preserve its pleiotropic physiological functions in normal cells, such as functions in immunity and inflammation [18, 20] Therefore, there is an urgent need to identify more effective therapeutic targets that regulate NF-κB in an appropriate manner as an alternative to global NF-κB blockade. Herein, we found that TRIM14 was over-expressed in TSCC and silencing TRIM14 significantly both inhibited the transcription activity of NF-κB and repressed the expression of various NF-κB target genes that are specifically regulate proliferation, apoptosis, metastasis and angiogenesis in TSCC, suggesting that TRIM14 could contribute to NF-κB activation and thereby represent a novel target for TSCC treatment.

The TRIM family proteins includes more than 70 members, which have been reported to be fundamentally involved in many biological processes, such as cell proliferation, apoptosis, angiogenesis, and invasion [21-23]. For example, TRIM29 has been reported to be upregulated in multiple tumor tissues and its overexpression can promote cancer development and progression [24-26]. Horn *et al.* showed that TRIM32 overexpression was coupled to the inhibition of TNF and ultraviolet (UV) irradiation-induced apoptosis, and that the upregulation of TRIM32 promoted UVB-induced squamous cell carcinomas proliferation, motility and transformation [27]. Additionally, TRIM68 was also significantly up-regulated in human prostate cancers and found to play an important role in prostate cancer progression [28]. Herein, we found that TRIM14 was up-regulated in TSCC and that TRIM14 overexpression promoted TSCC aggressiveness both *in vitro* and *in vivo*, which is in agreement with oncogenic-effect of TRIM family members. However, the mechanism of TRIM14 upregulation in TSCC remains unknown. Interestingly, we found that high levels of NF-κB could be recruited to the promoter region of TRIM14, according to ChIP sequencing tracks in the UCSC genome browser (http://genome.ucsc.edu/cgi-bin/hgGateway). Furthermore, according to The Cancer Genome Atlas (TCGA; http://cancergenome.nih.gov/), we found that TRIM14 amplification was positive in 26 (25%) of 104 cases, suggesting that the over-expression of TRIM14 in TSCC might be associated with genomic amplification. Thus, further studies of whether TRIM14 upregulation in TSCC could be attributed to genomic amplification or NF-κB mediated transcriptional up-regulation will be of great interest.

Interestingly, upon viral infection, TRIM14 can link NEMO to the mitochondrial antiviral signalosome and further activate the NEMO–IKK α–IKK β complex, which phosphorylates IκBα, to drive its ubiquitination and proteasomal, allowing the subsequently activation of NF-κB signaling [16]. Additionally, TRIM8 can modulate TNFα- and IL-1β-triggered NF-κB activation by mediating the K63-linked polyubiquitination of TAK1 in 293 cells [29]. Furthermore, TRIM22 has been reported to mediate NF-κB activation in a manner that is dependent on its N-terminal RING domain and C-terminal SPRY domain [30]. In accord with previous reports, our study showed that TRIM14 could activate the NF-κB signaling pathway in TSCC cells and TRIM14 levels were significantly correlated with NF-κB activity in clinical TSCC tissues. Interestingly, TRIM14 lacks the RING domain that can be found in other TRIM family members, which mediates activation of NF-κB signaling because of its E3 ubiquitin ligase activity. Therefore, elucidating the underlying mechanism whereby TRIM14 activates NF-κB signaling will require further investigation. Previously, it has been reported that the PRYSPRY domain of TRIM14 is critical for the interaction of TRIM14 with other proteins [31]. Consistently, we also found that TRIM14 ΔPRYSPRY failed to induce NF-κB activation, suggesting that the PRYSPRY domain of TRIM14 contribute to activation of NF-κB signaling (data not show). The precise mechanism in which molecular interacts with TRIM14 and involves in activation of inflammation signaling is under investigation currently in our laboratory.

In summary, we reported that TRIM14 was markedly upregulated in TSCC cells and clinical TSCC samples and that a positive correlation existed between TRIM14 expression and the prognosis of TSCC patients. Overexpression of TRIM14 augmented TSCC aggressiveness *in vitro* and *in vivo* and activated the NF-κB signaling pathway. Therefore, understanding the biological function of TRIM14 in TSCC progression both advance our knowledge of the mechanisms that underlie TSCC aggressiveness, and establish TRIM14 as a potential therapeutic target for the treatment of TSCC.

## MATERIALS AND METHODS

### Cell lines

Human TSCC cell lines SCC9, SCC25, Cal27 were purchased from American Type Culture Collection (ATCC, Manassas, VA, USA), TSCCA was purchased from the Chinese Academy of Medical Sciences Institute of Basic Medical Sciences and Tca8113 cells obtained from Shanghai Institute of Biochemistry and Cell Biology (SIBCB, Shanghai, China). All cancerous cell lines were grown in RPMI-1640 (Thermo Scientific, MA, USA) supplemented with 10% FBS (Hyclone, Rockford, IL, USA) and 100 units/ml penicillin and streptomycin. HIOEC was obtained from normal oral mucosa immortalized by transfection of HPV16 E6/E7 gene as previously described, and was cultured with defined keratinocyte medium-SFM (Gibco, USA). Two normal tongue epithelial cell lines (NTECs) was established by culturing normal tongue squamous epithelium from a non-tumor patient in keratinocyte/serum-free medium (Invitrogen Life Technologies, Carlsbad, CA, USA). All cell lines were maintained in a humidified incubator with 5% CO_2_ at 37°C.

### Patient information and tissue specimens

This study was conducted on a total of 116 archived TSCC samples, which were histopathologically and clinically diagnosed at the Sun Yat-sen University Cancer Center from 2001 to 2009. For the use of these clinical materials for research purposes, prior patient consent and approval from the Institutional Research Ethics Committee were obtained. Clinical information on the samples is summarized in Supplementary Table 1. Ten TSCC tissues and the matched adjacent noncancerous tissues were frozen and stored in liquid nitrogen until further use.

### Vectors, retroviral infection and transfection

A TRIM14 expression construct was generated by subcloning PCR-amplied full-length human TRIM14 cDNA into the pMSCV retrovirus plasmid, and human TRIM14-targeting short hairpin RNA (shRNA) oligonucleotides sequences were cloned into pSuper-retro-puro to generate pSuper-retro-TRIM14-RNAi(s). The shRNA sequences were: RNAi#1, TTCGTCAAGTAGTAATCTGAG; and RNAi#2, TTAAGGCGAATGTCCAATGGC (synthesized by Invitrogen). pNF-κB-luc and control plasmids (Clontech) were used to examine NF-κB activity. pBabe-Puro-IκBα-mut (plasmid#15291) expressing IκBα dominant-negative mutant (IκBα-mut) was purchased from Addgene (Cambridge, MA). Transfection of siRNA or plasmids was performed using the Lipofectamine 3000 reagent (Invitrogen, Carlsbad, CA) according to the manufacturer's instruction. Stable cell lines expressing TRIM14 or TRIM14 RNAi were selected for 10 days with 0.5μg/ml puromycin 48 h after infection.

### Western blotting analysis

Western blot was performed using anti-TRIM14 (Abcam, USA; 1:1000), anti-p-IκBα, IκBα and anti-p-IKKb, IKKb, anti-p65, anti-p84 antibodies (Cell Signaling, Danvers, MA, USA). The membranes were stripped and re-probed with an anti-α-tubulin antibody (Sigma, Saint Louis, MI) as a loading control.

### Chicken chorioallantoic membrane (CAM) assay

CAM assay was performed at day 6 of fertilized chicken eggs using a method previously described [32]. A 1.0-cm diameter window was opened on the egg shell (Yueqin Breeding Co. Ltd, Guangdong, China). The surface of the dermic sheet on the floor of the air sac was removed to expose the CAM. A 0.5-cm diameter filter paper was first placed on top of the CAM, and 100 μl conditioned medium was added onto the center of the paper. After the window was closed with sterile adhesive tape, the eggs were incubated at 37°C under 80–90% relative humidity for 4 days. Following fixation with stationary solution (methanol: acetone=1:1) for 15 min, the CAMs were cut and harvested, and gross photos of each CAM were taken with a digital camera (Panasonic, Osaka, Japan). The effect of conditioned media harvested from different cultured cells was evaluated by the number of second- and third-order vessels.

### HUVEC tube formation assay

Briefly, 200 μl of precooled Matrigel (Collaborative Biomedical Products) was transferred into each well of a 24-well plate and polymerized for 30 minutes at 37°C. HUVECs (2 × 10^4^) in 200 μl of conditioned medium were added to each well and incubated at 37°C, 5% CO_2_ for 20 hours. The capillary tube structure was photographed under a 100 × bright-field microscope, and quantified by measuring the total length of the completed tubes. Each condition was assessed in triplicate.

### Xenografted tumor model, IHC, and H&E staining

BALB/c-nu mice (4-5 weeks of age, 18-20 g) were purchased from the Center of Experimental Animal of Guangzhou University of Chinese Medicine. All experimental procedures were approved by the Institutional Animal Care and Use Committee of Sun Yat-sen University. The BALB/c nude mice were randomly divided into two groups (n = 6/group). One group of mice was inoculated subcutaneously with Cal127/Vector cells (5 × 10^6^) in the left dorsal flank and with Cal127/TRIM14 cells (5 × 10^6^) in the right dorsal flank per mouse. Another group was inoculated subcutaneously with Cal127/RNAi-vector cells (5 × 10^6^) in the left dorsal flank and with Cal127/TRIM14-RNAi cells (5 × 10^6^) in the right dorsal flank. Tumors were examined twice weekly; length and width measurements were obtained with calipers and tumor volumes were calculated using the equation (L*W2)/2. On day 40, tumors were detected by an IVIS imaging system, and animals were euthanized, tumors were excised, weighed and paraffin-embedded. Serial 6.0μm sections were cut and subjected to IHC analyzed using an anti-Ki67 and anti-CD31 antibodies (Dako, Glostrup, Denmark). Proliferation index was quantized by counting proportion of Ki67-positive cells. Apoptotic index was measured by percentage of TUNEL-positive cells.

### Luciferase assay

Cells (1 × 10^4^) were seeded in triplicate in 48-well plates and allowed to settle for 24 h. One hundred nanograms of luciferase reporter plasmids or the control plasmid, plus 1 ng of pRL-TK renilla plasmid (Promega), were transfected into cells using the Lipofectamine 3000 reagent (Invitrogen) according to the manufacturer's instruction. Luciferase and renilla signals were measured using the Dual Luciferase Reporter Assay Kit (Promega) according to a protocol provided by the manufacturer.

### Statistical analysis

Statistical tests for data analysis included Fisher's exact test, log-rank test, Chi-square test, and Student's 2-tailed t test. Multivariate statistical analysis was performed using a Cox regression model. Statistical analyses were performed using the SPSS 11.0 statistical software package. Data represent mean ± SD. *P* < 0.05 was considered statistically significant.

## SUPPLEMENTARY FIGURES AND TABLES



## References

[1] Jemal A, Siegel R, Ward E, Murray T, Xu J, Thun MJ (2007). Cancer statistics, 2007. CA: a cancer journal for clinicians.

[2] Ferlay J, Shin HR, Bray F, Forman D, Mathers C, Parkin DM (2010). Estimates of worldwide burden of cancer in 2008: Globocan 2008. International journal of cancer.

[3] Argiris A, Karamouzis MV, Raben D, Ferris RL (2008). Head and neck cancer. Lancet.

[4] Scully C, Bagan JV (2009). Recent advances in oral oncology 2008; squamous cell carcinoma imaging, treatment, prognostication and treatment outcomes. Oral oncology.

[5] Bello IO, Soini Y, Salo T (2010). Prognostic evaluation of oral tongue cancer: Means, markers and perspectives (ii). Oral oncology.

[6] Bello IO, Soini Y, Salo T (2010). Prognostic evaluation of oral tongue cancer: Means, markers and perspectives (i). Oral oncology.

[7] Loercher A, Lee TL, Ricker JL, Howard A, Geoghegen J, Chen Z, Sunwoo JB, Sitcheran R, Chuang EY, Mitchell JB, Baldwin AS, Van Waes C Nuclear factor-κb is an important modulator of the altered gene expression profile and malignant phenotype in squamous cell carcinoma. Cancer research.

[8] Wang Y, Lin Z, Sun L, Fan S, Huang Z, Zhang D, Yang Z, Li J, Chen W (2014). Akt/ezrin tyr353/nf-kappab pathway regulates egf-induced emt and metastasis in tongue squamous cell carcinoma. British journal of cancer.

[9] Yao J, Duan L, Fan M, Wu X (2006). Nf-kappab signaling pathway is involved in growth inhibition, g2/m arrest and apoptosis induced by trichostatin a in human tongue carcinoma cells. Pharmacological research:.

[10] Lu KW, Tsai ML, Chen JC, Hsu SC, Hsia TC, Lin MW, Huang AC, Chang YH, Ip SW, Lu HF, Chung JG (2008). Gypenosides inhibited invasion and migration of human tongue cancer scc4 cells through down-regulation of nfkappab and matrix metalloproteinase-9. Anticancer research.

[11] Peng B, Gu Y, Xiong Y, Zheng G, He Z (2012). Microarray-assisted pathway analysis identifies mt1x & nfkappab as mediators of tcrp1-associated resistance to cisplatin in oral squamous cell carcinoma. PloS one.

[12] Meroni G, Diez-Roux G (2005). Trim/rbcc, a novel class of ‘single protein ring finger’ e3 ubiquitin ligases. BioEssays: news and reviews in molecular, cellular and developmental biology.

[13] Nenasheva VV, Kovaleva GV, Khaidarova NV, Novosadova EV, Manuilova ES, Antonov SA, Tarantul VZ (2014). Trim14 overexpression causes the same transcriptional changes in mouse embryonic stem cells and human hek293 cells. *In vitro* cellular & developmental biology Animal.

[14] Kimsa MW, Strzalka-Mrozik B, Kimsa MC, Mazurek U, Kruszniewska-Rajs C, Gola J, Adamska J, Twardoch M (2014). Differential expression of tripartite motif-containing family in normal human dermal fibroblasts in response to porcine endogenous retrovirus infection. Folia biologica.

[15] Nenasheva VV, Kovaleva GV, Uryvaev LV, Ionova KS, Dedova AV, Vorkunova GK, Chernyshenko SV, Khaidarova NV, Tarantul VZ (2015). Enhanced expression of trim14 gene suppressed sindbis virus reproduction and modulated the transcription of a large number of genes of innate immunity. Immunologic research.

[16] Zhou Z, Jia X, Xue Q, Dou Z, Ma Y, Zhao Z, Jiang Z, He B, Jin Q, Wang J (2014). Trim14 is a mitochondrial adaptor that facilitates retinoic acid-inducible gene-i-like receptor-mediated innate immune response. Proceedings of the National Academy of Sciences of the United States of America.

[17] Hoesel B, Schmid JA (2013). The complexity of nf-kappab signaling in inflammation and cancer. Molecular cancer.

[18] DiDonato JA, Mercurio F, Karin M (2012). Nf-kappab and the link between inflammation and cancer. Immunological reviews.

[19] He D, Xu Q, Yan M, Zhang P, Zhou X, Zhang Z, Duan W, Zhong L, Ye D, Chen W (2009). The nf-kappa b inhibitor, celastrol, could enhance the anti-cancer effect of gambogic acid on oral squamous cell carcinoma. BMC cancer.

[20] Staudt LM (2010). Oncogenic activation of nf-kappab. Cold Spring Harbor perspectives in biology.

[21] Hatakeyama S (2011). Trim proteins and cancer. Nature reviews Cancer.

[22] Jiang T, Tang HM, Lu S, Yan DW, Yang YX, Peng ZH (2013). Up-regulation of tripartite motif-containing 29 promotes cancer cell proliferation and predicts poor survival in colorectal cancer. Medical oncology.

[23] Cambiaghi V, Giuliani V, Lombardi S, Marinelli C, Toffalorio F, Pelicci PG (2012). Trim proteins in cancer. Advances in experimental medicine and biology.

[24] Kosaka Y, Inoue H, Ohmachi T, Yokoe T, Matsumoto T, Mimori K, Tanaka F, Watanabe M, Mori M (2007). Tripartite motif-containing 29 (trim29) is a novel marker for lymph node metastasis in gastric cancer. Annals of surgical oncology.

[25] Hawthorn L, Stein L, Panzarella J, Loewen GM, Baumann H (2006). Characterization of cell-type specific profiles in tissues and isolated cells from squamous cell carcinomas of the lung. Lung cancer.

[26] Santin AD, Zhan F, Bellone S, Palmieri M, Cane S, Bignotti E, Anfossi S, Gokden M, Dunn D, Roman JJ, O'Brien TJ, Tian E, Cannon MJ, Shaughnessy J, Pecorelli S (2004). Gene expression profiles in primary ovarian serous papillary tumors and normal ovarian epithelium: Identification of candidate molecular markers for ovarian cancer diagnosis and therapy. International journal of cancer.

[27] Horn EJ, Albor A, Liu Y, El-Hizawi S, Vanderbeek GE, Babcock M, Bowden GT, Hennings H, Lozano G, Weinberg WC, Kulesz-Martin M (2004). Ring protein trim32 associated with skin carcinogenesis has anti-apoptotic and e3-ubiquitin ligase properties. Carcinogenesis.

[28] Miyajima N, Maruyama S, Bohgaki M, Kano S, Shigemura M, Shinohara N, Nonomura K, Hatakeyama S (2008). Trim68 regulates ligand-dependent transcription of androgen receptor in prostate cancer cells. Cancer research.

[29] Li Q, Yan J, Mao AP, Li C, Ran Y, Shu HB, Wang YY (2011). Tripartite motif 8 (TRIM8) modulates TNFα- and IL-1β- triggered NF-κB activation by targeting TAK1 for K63- linked polyubiquitination. Proceedings of the National Academy of Sciences of the United States of America.

[30] Yu S, Gao B, Duan Z, Xu W, Xiong S (2011). Identification of tripartite motif-containing 22 (trim22) as a novel nf-kappab activator. Biochemical and biophysical research communications.

[31] Zhou Z, Jia X, Xue QH, Dou ZX, Ma YJ, Zhao ZD, Jiang ZF, He B, Jin Q, Wang JW (2014). Trim14 is a mitochondrial adaptor that facilitates retinoic acid-inducible gene-i-like receptor-mediated innate immune response. Proceedings of the National Academy of Sciences of the United States of America.

[32] Celerier J, Cruz A, Lamande N, Gasc JM, Corvol P (2002). Angiotensinogen and its cleaved derivatives inhibit angiogenesis. Hypertension.

