# Supplementary material for: Overexpression of TRIM14 promotes tongue squamous cell carcinoma aggressiveness by activating the NF-κB signaling pathway
Source: Oncotarget. 2016 Jan 18;7(9):9939–50. doi: 10.18632/oncotarget.6941 (PMC4891094; doi:10.18632/oncotarget.6941)
Supplement: Supplementary file 1 [file oncotarget-07-09939-s001.pdf]

SUPPLEMENTARY FIGURES AND TABLES

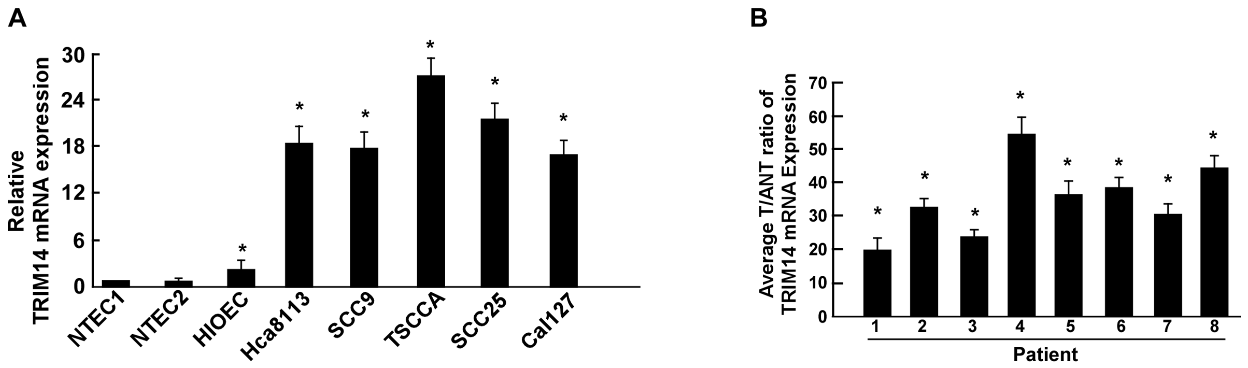

Supplementary Figure S1: mRNA expression analysis shows that TRIM14 was up-regulated in TSCC cell lines and tissues. A. Real-time PCR analysis of TRIM14 expression in two normal tongue epithelial cells, one immortalized oral epithelial cell line (HIOEC) and in TSCC cell lines (Hca8133, SCC9, TSCCA, SCC25 and Cal127). Transcript levels were normalized to *GAPDH* expression. **B.** Real-time PCR analysis of TRIM14 expression in TSCC tissues (T) with matched adjacent non-tumor tissues (N) from 8 patients. Transcript levels were normalized to *GAPDH* expression. Each bar represents the mean ± SD of three independent experiments. *$P < 0.05$.

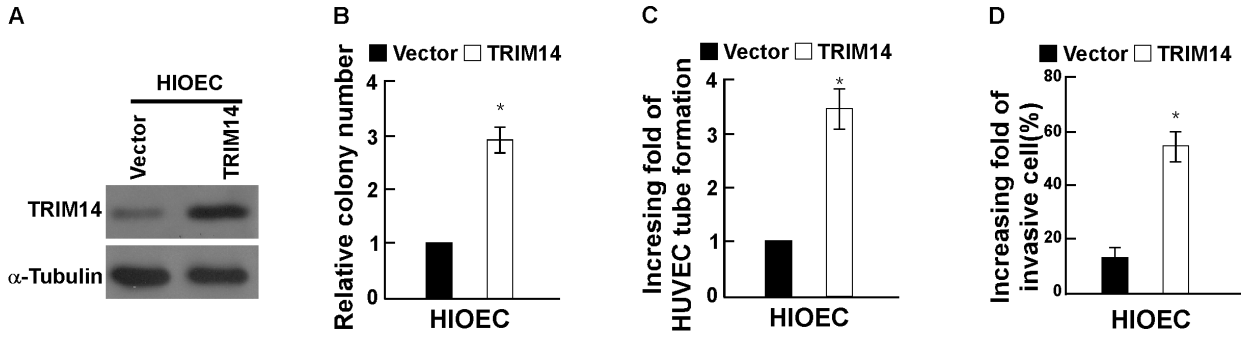

Supplementary Figure S2: Up-regulation of TRIM14 expression promotes immortalized oral epithelial cell line (HIOEC) cell aggressiveness *in vitro*. A. Western blot analysis of TRIM14 expression in HIOEC cells. α-Tubulin was used as a loading control. **B.** Representative pictures (left panel) and quantification (right panel) of the colony numbers of indicated cells as determined using an anchorage-independent growth assay. Colonies larger than 0.1 mm in diameter were scored. **C.** Representative images (left panel) and quantification (right panel) of HUVECs cultured on matrigel-coated plates with conditioned medium from vector control and TRIM14-transduced TSCC cells. **D.** Representative pictures (left panel) and quantification (right panel) of invaded cells were analyzed using a transwell matrix penetration assay. * P <0.05.

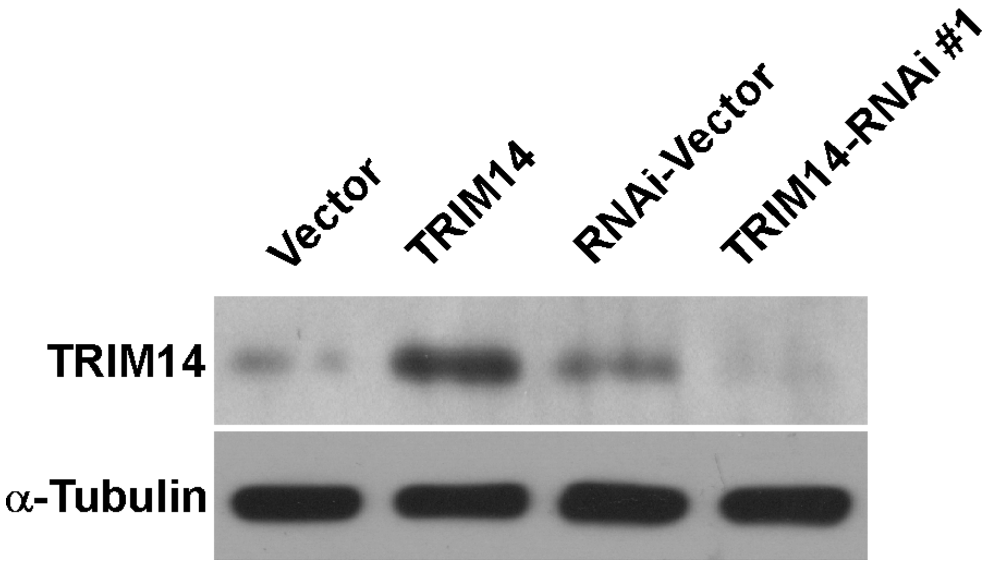

Supplementary Figure S3: Western blot analysis of TRIM14 in the indicated xenografts tumors.

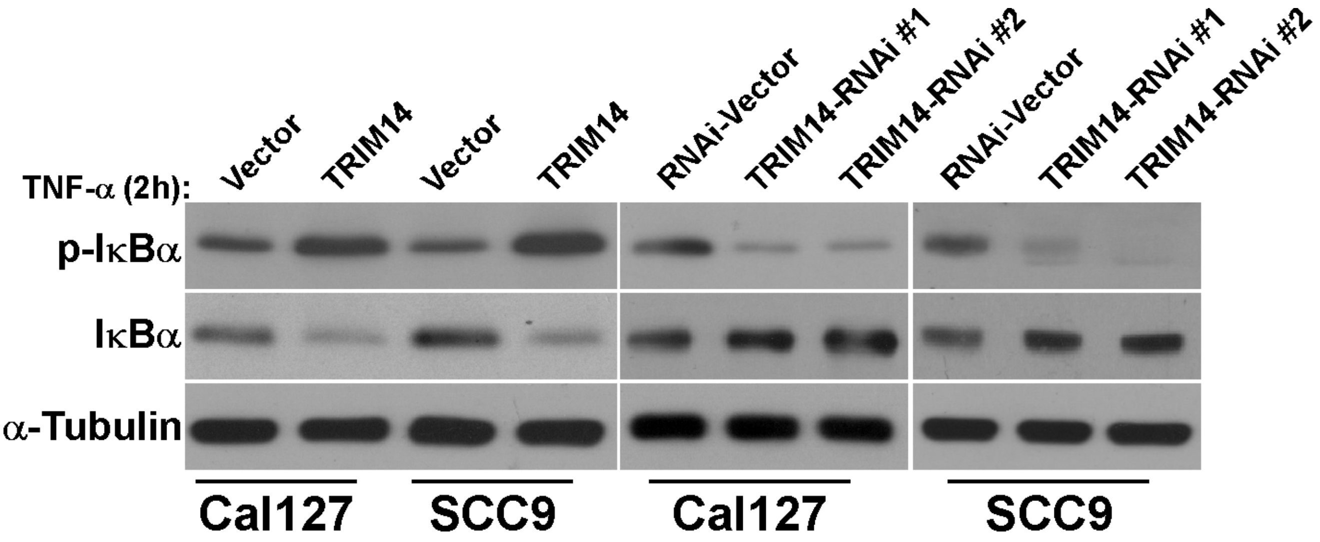

Supplementary Figure S4: The effect of TRIM-14 on induction of IκBα degradation. Western blotting analysis of the expression levels of IκBα and p-IκBα was examined in the cells upon TNF-α treatment at 2hr. α-tubulin was used as a loading control.

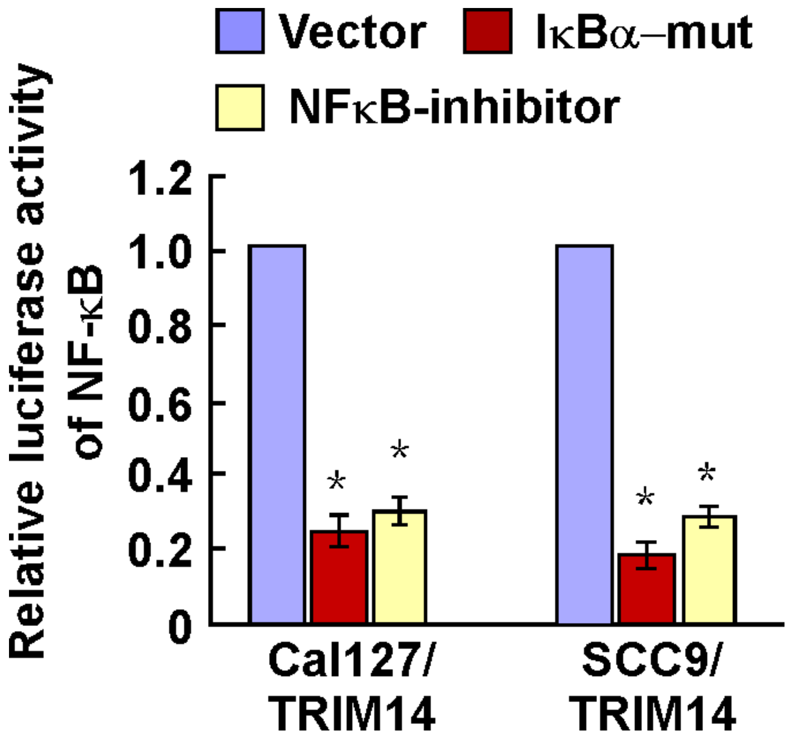

Supplementary Figure S5: Relative of luciferase reporter activity in the indicated TSCC cells transfected with vector or IκBα-mut or treated with an NF-κB inhibitor (JSH-23).

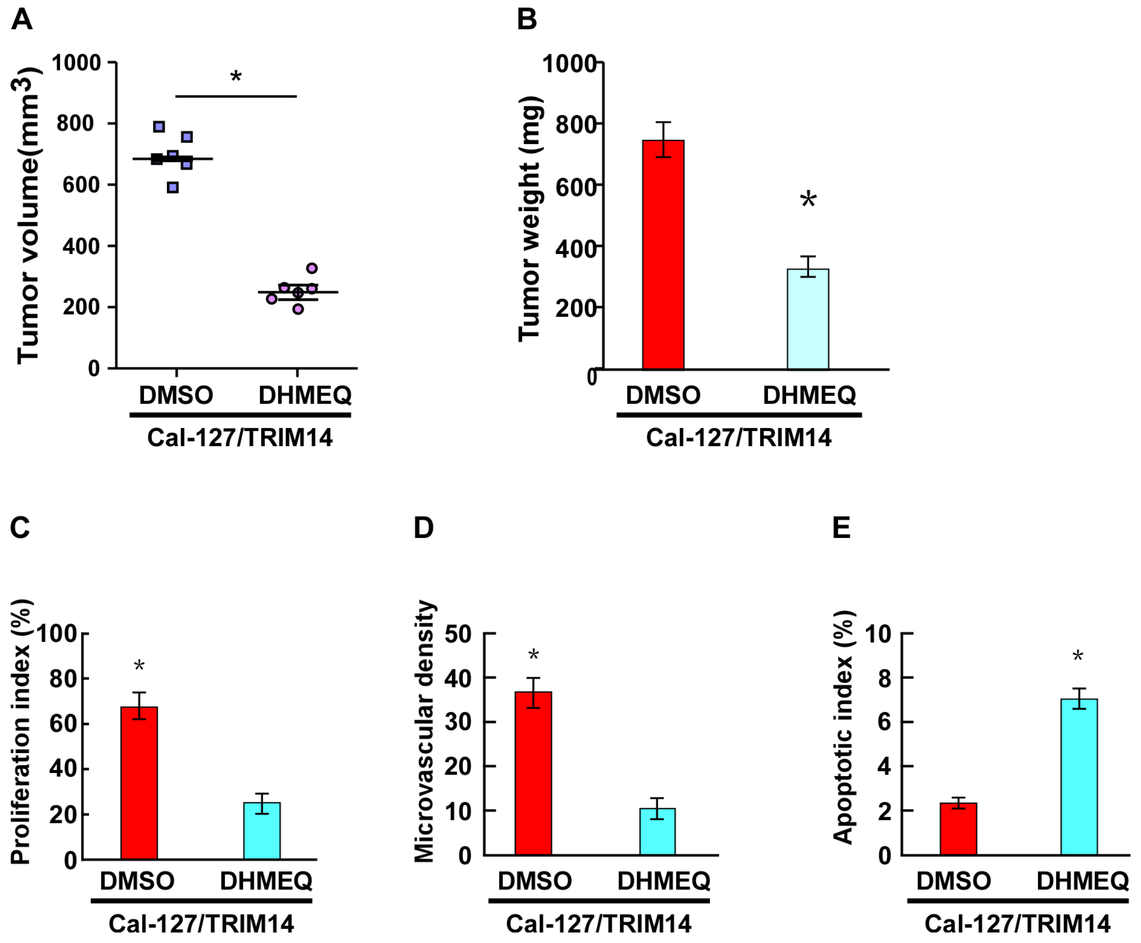

Supplementary Figure S6: Blockade of NF-κB pathway attenuates TRIM14-dependent cancer cell progression *in vivo*. A. Tumor volumes were measured. **B.** Mean tumor weights. **C-E.** IHC staining demonstrated the expression of Ki67 and CD31 as well as TUNEL-positive cells in the indicated tissues.

Supplementary Table S1: Clinicopathological characteristics of studied patients and expression of TRIM14 in TSCC

Factor	NO.	(%)
**Gender**		
Male	53	45.6
Female	63	54.4
**Age (years)**		
≤50	56	48.3
>50	60	51.7
**Clinical stage**		
I	26	22.4
II	36	31.0
III	29	25.0
IV	25	21.6
**T classification**		
T_1	25	21.6
T_2	37	31.8
T_3	32	27.6
T_4	22	19.0
**N classification**		
N_0	50	43.1
N_1	66	56.9
**M classification**		
No	54	46.6
Yes	62	53.4
**Vital status**		
Alive	52	44.8
Dead	64	55.2
**Expression of TRIM14**		
Low expression	57	46.6
High expression	59	53.4

Supplementary Table S2: Correlation between the clinicopathological features and expression of TRIM14

Patient characteristics		TRIM14 expression		*P*-value
		Low or none	High	
**Gender**	Male	23	30	0.270
	Female	34	29	
**Age (years)**	≤50	27	33	0.457
	>50	30	26	
**Clinical stage**	I	19<br>132<br>62	7<br>44	0.009
	II	18	18	
	III	14	15	
	IV	6	19	
**T classification**	T_1	20<br>132<br>62	5<br>44	0.01
	T_2	17	20	
	T_3	12	20	
	T_4	8	14	
**N classification**	N_0	37<br>204	17<br>0	< 0.001
	N_1	20	42	
**M classification**	No	41	19	< 0.001
	Yes	16	40	
**Vital status**	Alive	42	22	< 0.001
	Dead	15	37	

Supplementary Table S3: Univariate and multivariate analysis of different prognostic parameters in patients with TSCC by Cox-regression analysis

	Univariate analysis		Multivariate analysis	
	P	**Hazard ratio (95% CI)**	***P***	**Hazard ratio-RR (95% CI)**
**Clinical stage**<br>I<br>II<br>III<br>IV	0.002	1.567<br>(1.171-2.096)	0.015	1.677<br>(1.286-2.188)
**M classification**<br>M_0<br>M_1	0.003	1.703<br>(1.069-2.714)	0.013	5.277<br>(3.771-7.385)
**N classification**<br>N_0<br>N_1	0.002	2.221<br>(1.327-3.717)	0.020	1.514<br>(1.314-1.841)
**TRIM14 expression**<br>Low expression<br>High expression	0.01	3.744<br>(1.713-8.183)	0.031	1.879<br>(1.178-2.998)

Supplementary Table S4: Correlation between related gene expression and TRIM14 level in TSCC samples

Characteristics		TRIM14 expression		*P*-value
		Low	High	
**p65 expression**	Low	20<br>204	32<br>0	= 0.042
	High	37	27	
**p-IκBα expression**	Low	40	25	= 0.003
	High	17	34	
**p-IKKβ**	Low	33	21	= 0.025
	High	24	38	
**Ki67 signals**	Low	39	22	= 0.001
	High	18	37	
**TUNEL positive signals**	Low	20	38	= 0.003
	High	37	21	
